# Enhancer networks revealed by correlated DNAse hypersensitivity states of enhancers

**DOI:** 10.1093/nar/gkt374

**Published:** 2013-05-21

**Authors:** Justin Malin, Mohamed Radhouane Aniba, Sridhar Hannenhalli

**Affiliations:** ^1^Center for Bioinformatics and Computational Biology, University of Maryland, College Park, MD, 20740, USA, ^2^Computational Biology, Bioinformatics, and Genomics Program, University of Maryland, College Park, MD, 20740, USA and ^3^Department of Cell Biology and Molecular Genetics, University of Maryland, College Park, MD, 20740, USA

## Abstract

Mammalian gene expression is often regulated by distal enhancers. However, little is known about higher order functional organization of enhancers. Using ∼100 K P300-bound regions as candidate enhancers, we investigated their correlated activity across 72 cell types based on DNAse hypersensitivity. We found widespread correlated activity between enhancers, which decreases with increasing inter-enhancer genomic distance. We found that correlated enhancers tend to share common transcription factor (TF) binding motifs, and several chromatin modification enzymes preferentially interact with these TFs. Presence of shared motifs in enhancer pairs can predict correlated activity with 73% accuracy. Also, genes near correlated enhancers exhibit correlated expression and share common function. Correlated enhancers tend to be spatially proximal. Interestingly, weak enhancers tend to correlate with significantly greater numbers of other enhancers relative to strong enhancers. Furthermore, strong/weak enhancers preferentially correlate with strong/weak enhancers, respectively. We constructed enhancer networks based on shared motif and correlated activity and show significant functional enrichment in their putative target gene clusters. Overall, our analyses show extensive correlated activity among enhancers and reveal clusters of enhancers whose activities are coordinately regulated by multiple potential mechanisms involving shared TF binding, chromatin modifying enzymes and 3D chromatin structure, which ultimately co-regulate functionally linked genes.

## INTRODUCTION

Eukaryotic transcription is intricately regulated at multiple levels, including epigenomic modifications, chromatin reorganization and sequence-specific binding of transcription factor (TF) to either proximal promoter regions or to distal enhancer/repressor regions of a gene (1,2). Distal enhancers can regulate their target genes from long distances, the most extreme case being the Shh gene’s enhancer at ∼1 Mb away, and are especially important in regulating critical developmental genes (3,4). Recent advances in sequencing technologies have revealed that cell-specific enhancers are often marked by P300 binding (a histone acetyltransferase and transcription coactivator) (5,6), as well as other epigenomic marks such as DNAse hypersensitivity (DHS), H3K4me1, H3K27ac and so forth. (7,8). Various combinations of these marks have been used to generate genome-wide catalogs of potential cell-type-specific distal enhancers (9). However, the target genes of the distal enhancers remain unknown for the most part. Moreover, the mechanisms by which distal enhancers regulate the expression of their target genes are not completely understood.

Functionally linked genes, e.g. components of a biological pathway or a protein complex, tend to be co-expressed and are presumed to be co-regulated (10–13). Gene networks based on co-expression patterns of gene pairs across multiple conditions and/or cell types reveal intricate organization of genes into pathways and functional groups (14). Similar to functionally related genes, functionally related enhancers, i.e. those regulating functionally related genes, share TF binding sites and are likely to have spatio-temporal coordinated activity (15). A network-level analysis of coordinated activities of distal enhancers has not been reported, and such an analysis is likely to reveal higher order organization of a global transcriptional regulatory network mediated by distal enhancers. Analogous to using expression level to quantify transcriptional activity of a gene, DHS of an enhancer region has been proposed as a proxy for its condition-specific regulatory activity (8,16,17). Under the encyclopedia of genomic elements (ENCODE) project, whole-genome DHS profiles have been generated for dozens of human cell types (18). Analogous to using cross-condition expression correlation to infer gene networks, cross-condition DHS correlation can be used to infer enhancer networks. Indeed, a recent report has shown the effectiveness of using cross-condition DHS correlation between distal enhancers and gene promoters to identify distal enhancers of genes (19).

Tissue-specific enhancers are often marked by P300 binding. Most of the tested P300 bound regions in mouse embryonic forebrain, midbrain and limb tissue were shown to function as enhancers in transgenic mice (5). Thus, a genome-wide profile of P300 bound regions provides a reasonable approximation for candidate enhancer regions. Starting with ∼100 000 P300 bound regions in one or more of four cell types as candidate enhancers, here we perform a detailed network-level analysis of enhancers based on their DHS correlation across 72 cell types. We identified a large set of enhancer pairs whose DHS level was significantly correlated across cell types, even after controlling for autocorrelation of DHS along the chromosome. We found that (i) correlated enhancers tend to share common TF binding motifs. (ii) Several chromatin modification enzymes (CME) preferentially interact with TFs whose binding sites co-occur in pairs of correlated enhancers. (iii) Presence of shared motifs can discriminate between correlated and uncorrelated enhancer pairs with 73% accuracy. (iv) Using the gene closest to an enhancer as its putative target, we found that the targets of correlated enhancers have correlated expression and are involved in common biological processes. (v) Based on Hi-C data on chromatin spatial interaction in two different cell types, we found that correlated enhancers are spatially proximal significantly more often than expected. (vi) Strong enhancers, those with higher expression levels of the nearest gene, tend to be correlated with fewer enhancers than weak enhancers but preferentially correlate with other strong enhancers, whereas weak enhancers are correlated with a greater number of enhancers and preferentially correlate with other weak enhancers. (vii) We constructed enhancer networks based on correlated activity and shared TF motifs, and found significant enrichment of specific biological processes among the putative gene targets of the enhancer modules.

Overall, our analysis suggests that functionally linked genes may be co-regulated by distal enhancers whose activities are regulated by common sets of TFs and mediated by both 3D chromatin structure as well as CMEs. Our work represents the first investigation of enhancer networks based on correlated activity across multiple cell types.

## MATERIALS AND METHODS

### P300 and DHS data overview

P300 binding has been shown to be a reliable marker of tissue specific enhancers (5). As a starting set of candidate enhancers, we extracted from Gene Expression Omnibus (GEO) (20) the genomic regions bound by P300 in at least one of the four cell types—HepG2 (GEO accession Id GSM758575), GM12878 (GEO Id GSM803387), H1-HESC (GEO Id GSM803542) and SK-N-SH_RA (GEO Id GSM803495). For each of the four data sets, we extracted the P300 peaks and, in case of overlaps, used the center of merged overlapping regions. We thus obtained 98 353 enhancer regions, with an average length of 500 bp centered at the center of the P300 peaks, <5% (7%) of which overlap with 2 kb (5 kb) upstream of annotated ENSEMBL transcripts. From the ENCODE database (18), we extracted the genome-wide DHS broad peak data for each of the 72 tissue types represented; for tissue types with more than one data set available, we chose the set with the greatest number of peaks. For each enhancer, with respect to each tissue, DHS was set to 1 if the 500 bp enhancer region overlapped a DHS peak; otherwise, it was set to 0. This procedure yielded a 98 353 × 72 binary matrix, with rows corresponding to enhancers, columns to tissue (or cell) types and matrix entries reflecting the ‘activity state’ of an enhancer in a tissue. To minimize dependencies, tissues were clustered based on similarity, into 37 clusters, including 25 singletons (Supplementary Table S1), and only the most representative tissue from each cluster was retained for further analyses. Accordingly, the DHS matrix was reduced from 72 columns to 37.

### Mutual information

Mutual information (MI) between two binary vectors X and Y is defined as

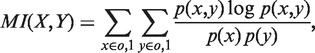

where p(x) is the probability of x in X, p(y) is probability of y in Y and p(x,y) is the joint probability that x and y co-occur in vectors X and Y. Informally, MI quantifies how much knowing one of the two vectors helps determine the other. Relative advantages of using MI over other measures such as correlation have been discussed previously, e.g. (21).

### Controlling for DHS autocorrelation

**Figure 1.**
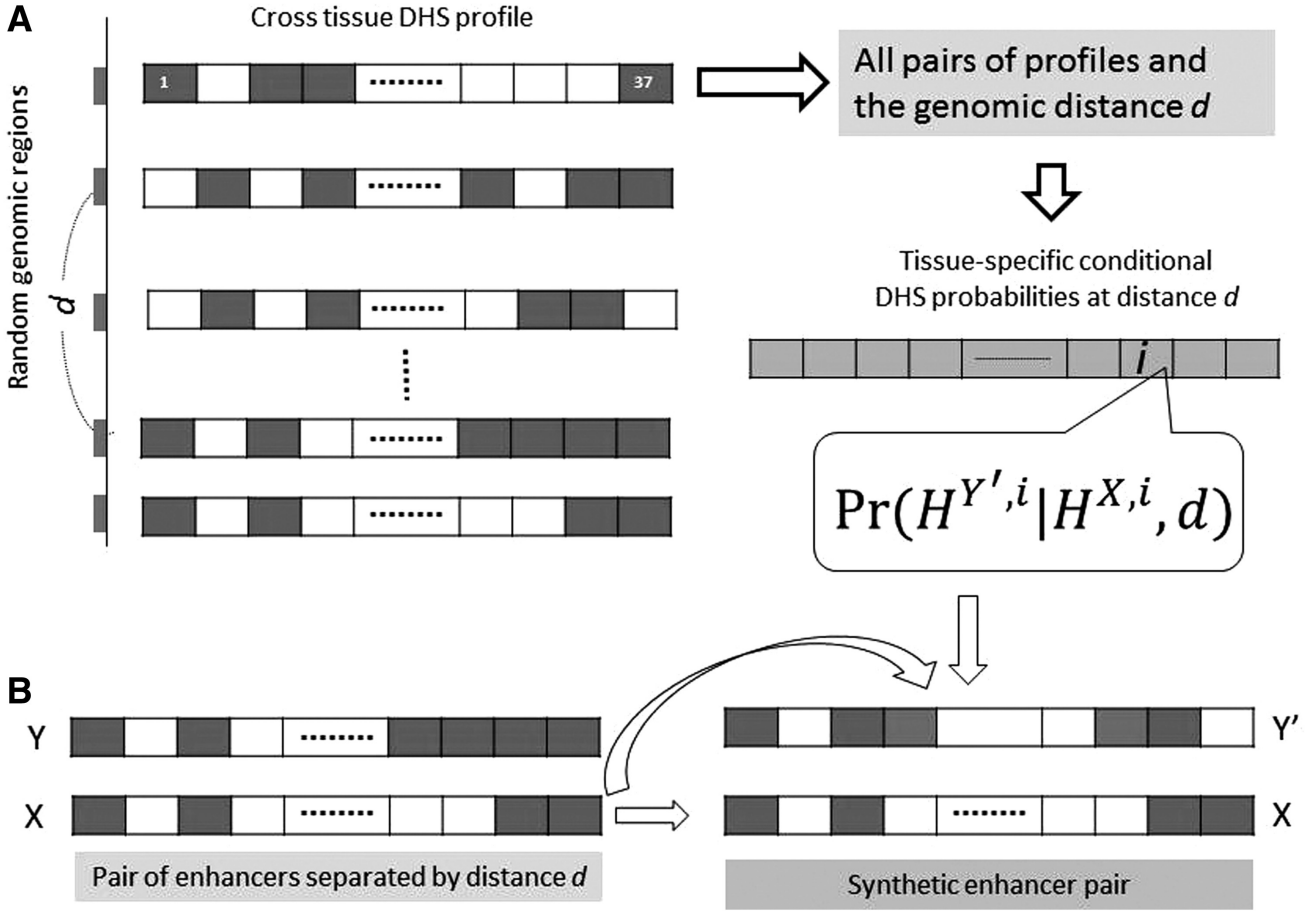
Generating the synthetic enhancer data to account for autocorrelation. (**A**) Starting with a large set of random genomic regions and their DHS profiles across 37 cell types, we estimated, for each cell type separately, the conditional probability of observing DHS at a location *Y*′, given the DHS status at another location *X* at distance *d* from *X*. (**B**) Given a pair of enhancer DHS profiles (*X,Y*), we generate a synthetic pair of DHS profiles as (*X,Y′*) where *Y′* is randomly generated from *X* and the conditional probabilities estimated in (A). See text for further details. Blue: DHS = 1 (open chromatin); white: DHS = 0 (closed chromatin).

**Figure 2.**
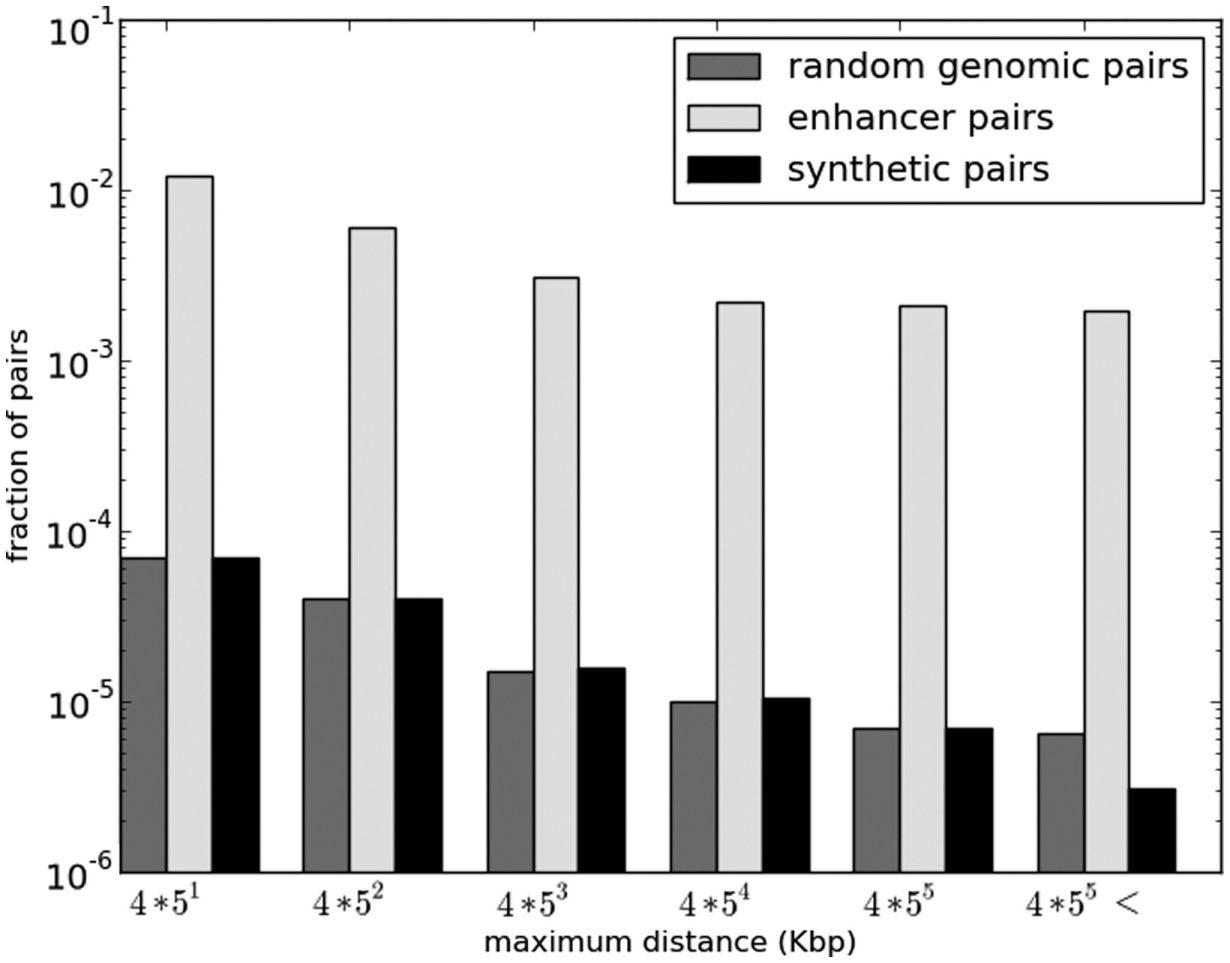
MI of chromatin states is higher among enhancer pairs than background pairs, and it decreases monotonically with increasing distance. Plot shows the relationship between inter-enhancer genomic distance and the number of actual and synthetic enhancer pairs with MI above 0.4 across 37 representative cell types. Enhancer pairs (light gray) were selected from 98 000 enhancers identified based on P300 ChIP-Seq peaks by exhaustively pairing all enhancers sharing the same chromosome and <12.5 Mb apart. Five million additional pairs were sampled for distances >12.5 Mb, as well as 1 million inter-chromosomal pairs. As a negative control, the DHS vector of a randomly chosen member of each enhancer pair was used as a seed to generate a paired synthetic DHS vector by conditioning on observed cell type-specific DHS autocorrelation along the genome. This resulted in 1 synthetic enhancer pair (black) for each enhancer pair; pairs of random genomic segments (gray) were generated in the same fashion as enhancer pairs by drawing from 100 000 random genomic segments of mean length 500 bp. MI of 0.4 roughly corresponds to FDR 0.01 (see text).

We controlled for the observed cell-type-specific DHS autocorrelation to detect significantly correlated enhancer pairs (Figure 1). Separately for each of the 37 cell types, based on 100 000 random genomic segments, we estimated the autocorrelation probability of DHS at a location conditional on DHS at another location at a specific distance-range (or distance-bin). In particular, given a cell type, enhancer **X**, and enhancer **Y** at distance-bin *d* from **X**, we estimate the probability that **Y** is DHS conditional on the DHS status of X. This tissue-specific and distance-specific autocorrelation probability was then used to create a ‘synthetic’ enhancer pair corresponding to each of the actual enhancer pairs. Each synthetic pair consists of the DHS vector for one member of the actual pair and a randomly generated vector of 37 binary DHS values replacing the other member (Figure 1). The autocorrelation conditional probabilities estimated above are used to generate the synthetic vector, conditioned on cell type and distance-bin. As a consequence, DHS data for synthetic pairs preserve for each tissue type both the mean DHS and extent of autocorrelation observed in the real genome, resulting in an MI profile that is virtually identical to that of random genomic segment pairs (Figure 2).

### TF binding site identification

For each enhancer sequence and each of the 981 positional weight matrix for vertebrate TFs in transcription factor binding sites (TRANSFAC) database (22), we used our previously published tool (23) to identify binding sites based on a score threshold of 95th percentile. For each enhancer, only presence/absence of a motif was noted.

### Motif co-occurrence score

We quantified the tendency of each motif to co-occur in correlated pairs of enhancers relative to its expected co-occurrence frequency, assuming independent occurrence of motifs among enhancers. If p represents the fraction of enhancers in which a motif occurs then assuming independence the motif is expected to co-occur in *p*^2^ of the enhancer pairs. The motif co-occurrence score is defined as the ratio of the observed co-occurrence frequency and the expected frequency *p*^2^.

### Removing dependencies among pairs

In both the foreground and the background, transitive dependencies were removed; enhancer pairs were excluded if either of the enhancers was part of a previously included pair. In addition, we ensured that the distribution of inter-enhancer distances was identical for the foreground and the background.

### Motif clustering

Motifs were clustered based on similarity owing to structural similarities between the corresponding TFs. All pairwise motif similarity scores for the 981 vertebrate motifs were obtained from the author of the STAMP DNA-binding motif comparison tool (24). Using pairwise similarity, the motifs were hierarchically clustered using the ‘hierarchy’ module in SciPy’s ‘cluster’ package (www.scipy.org) for Python based on Euclidean distance and complete linkage. The resulting tree was trimmed using the module’s ‘fcluster’ function with a maximum co-phenetic distance criterion that produced 142 disjoint clusters.

### Tissue clustering

We computed the pairwise similarity between tissues based on their genome-wide DHS profiles for all enhancers. We used the ‘linkage’ method in Scipy’s ‘hierarchy.cluster’ class to perform hierarchical clustering based on average linkage in combination with Russell–Rao pairwise distance (i.e. the fraction of enhancers with a DHS state of one in the two tissues). The resulting tree was trimmed using the class’s *fcluster* method and with an inconsistency criterion that resulted in 37 clusters, including 25 singletons. In each cluster of size 3 or larger, the tissue with the lowest mean distance to other cluster members was retained, whereas in clusters of size 2, it was the tissue with the greatest mean separation from all other tissues in the sample.

### Determination of concordance between enhancer cluster’s and target gene cluster’s tissue-specific activity

We clustered the 84 tissue types in the CTen database and the 72 cell/tissue types in the DHS database into 34 and 23 cytologically motivated classes, respectively. [Class sizes ranged from 1 to 19 (brain) for CTen tissues and 1–15 (endothelium and blood) for DHS cell types]. Agreement in tissue-specific activity was assessed based on the 17 classes shared between the two domains; tissues falling outside of these classes were not considered. For each target gene cluster, we first identified the tissue in which the genes exhibit tissue-specific activity according to CTen [False Discovery Rate (FDR) 0.01]. Then, we obtained the corresponding tissue class in the DHS data set and determined the rank of that tissue class for the corresponding enhancer cluster activity as follows. For an enhancer cluster, and for each tissue class, we determine the ratio between (i) the fraction of enhancers in the particular cluster having DHS in that tissue class and (ii) the fraction of ‘all’ enhancers with DHS in that tissue class. We then use this tissue-specific fold enrichment to rank all 23 tissue classes. We are interested in the rank of the specific tissue class in which the corresponding genes had robust and specific activity according to CTen. We thus obtain a rank for each cluster, and we determined the median rank among all clusters in a clustering. We applied eight different clustering parameters and for each clustering obtained the median rank for the actual clusters as well as for randomly generated background clusters with same size. Finally, we compared the median ranks for the foreground and background clusters using paired Wilcoxon test.

## RESULTS

### Data overview

P300 binding has been shown to be a reliable marker of tissue specific enhancers (5). As a starting set of candidate enhancers, we obtained 98 353 P300 peaks in four different cell types (see ‘Materials and Methods’ section). We extracted genome-wide DHS broad peak data for 72 tissue types in the ENCODE database (18) and clustered the 72 tissues into 37 representatives (Supplementary Table S1) based on genome-wide correlation (see ‘Materials and Methods’ section). Enhancers vary broadly (0–37 tissues) in the number of tissues in which they overlap a DHS peak (see distribution in Supplementary Figure S1). For each enhancer, we constructed a DHS profile as a binary vector of length 37 corresponding to 37 cell types, by setting the DHS value to 1 if the enhancer region overlapped a DHS peak in the particular tissue; otherwise, it was set to 0. This procedure yielded a 98 353 × 37 enhancer ‘activity’ matrix, with rows corresponding to enhancers, columns to tissue (or cell) types.

### Identifying enhancers with correlated activity

**Figure 3.**
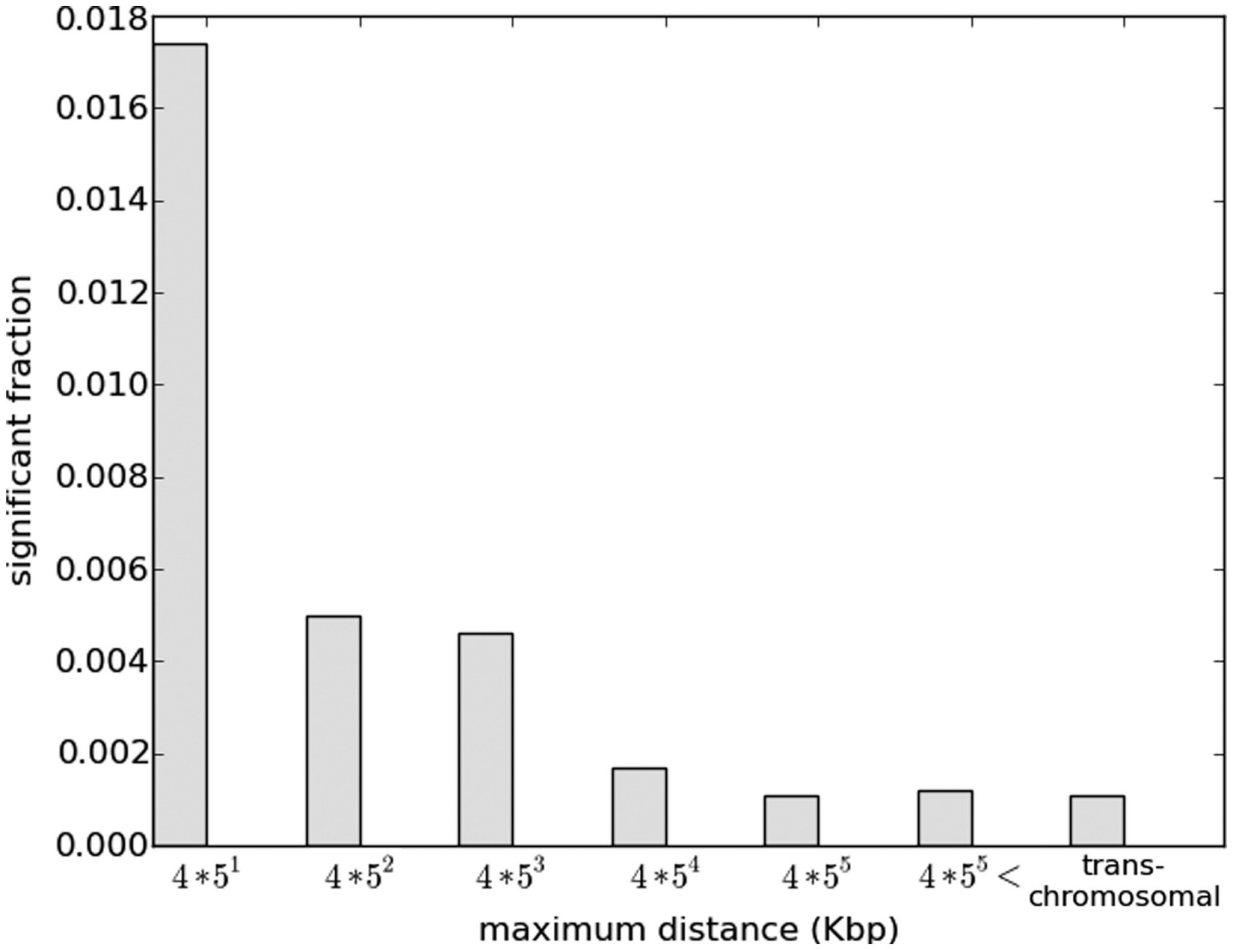
Chromatin states of a large number of enhancer pairs are significantly correlated. The plot shows the fraction of pairs with significant MI as a function of inter-enhancer distance. Significant enhancer pairs were identified by setting a threshold MI for each bin that corresponded to a nominal false discover rate of 0.1% (see text). The plot is based on significant pairs after greedily removing pairs inducing transitive relationships. The percentage of significant enhancer pairs drops with pairwise distance but stabilizes at ∼2 Mb. Moreover, if one of the enhancers in our set overlapped both with a strong and weak chromHMM enhancer, we excluded that enhancer as well as the overlapping chromHMM enhancers from our calculations.

We quantified correlated activity for a pair of enhancers using the information theoretic measure *MI* using DHS in 37 tissues (see ‘Materials and Methods’ section). However, MI can be biased toward enhancer pairs that are near each other on the genome, if DHS regions are long or tend to cluster on the genome. We tested this by selecting intra-chromosomal pairs using 100 000 random genomic segments and computing their MI. Figure 2 shows that the fraction of segment-pairs with MI >0.4 decays monotonically with increasing inter-segment distance, suggesting autocorrelation of DHS along the genome; the same trend holds for other MI thresholds. The same trend also holds for the 35 million enhancer pairs tested, but crucially, the fraction of enhancer pairs with high MI is greater than that of random genomic segments (represented by yellow and gray bars, respectively, in Figure 2). We controlled for the observed cell-type-specific DHS autocorrelation to detect significantly correlated enhancer pairs (see ‘Materials and Methods’ section and Figure 1). We consider six distance-bins ranging from 20 Kb to 12.5 Mb (Figure 3) and within each distance-bin, we identify significantly correlated enhancer pairs by estimating a nominal FDR (25) by comparing MI scores for actual and control pairs (see ‘Materials and Methods’ section).

### A sizable fraction of enhancer pairs has correlated activity across cell types

We exhaustively assessed ∼35 million intra-chromosomal enhancer pairs separated by <12.5 Mb; additional sampling at larger distances and across chromosomes suggested that 12.5 Mb ceiling is sufficient to capture general patterns. Despite distance bin-specific FDR control, the fraction of enhancers that are significantly correlated declines with increasing distance (Figure 3); after removing transitive relationships (‘Materials and Methods’ section), at FDR of 0.1%, the fraction decreases from 1.7% pairs at 20 Kb to 0.1% for pairs separated by >12.5 Mb. The corresponding fractions at 5% FDR are 4.8–1.3%. A similar trend is also observed when background pairs are pooled across distance bins and a single FDR test is conducted (Supplementary Figure S2a). Similarly, these trends are preserved when we used random trans-chromosomal enhancer pairs as the background to calculate the FDR (Supplementary Figure S2b). Across all bins, at an FDR of 1%, we detect a total of 313 757 significant enhancer pairs, covering 32% of enhancers.

### Strong and weak enhancers have different degrees of connectivity and are assortative

**Figure 4.**
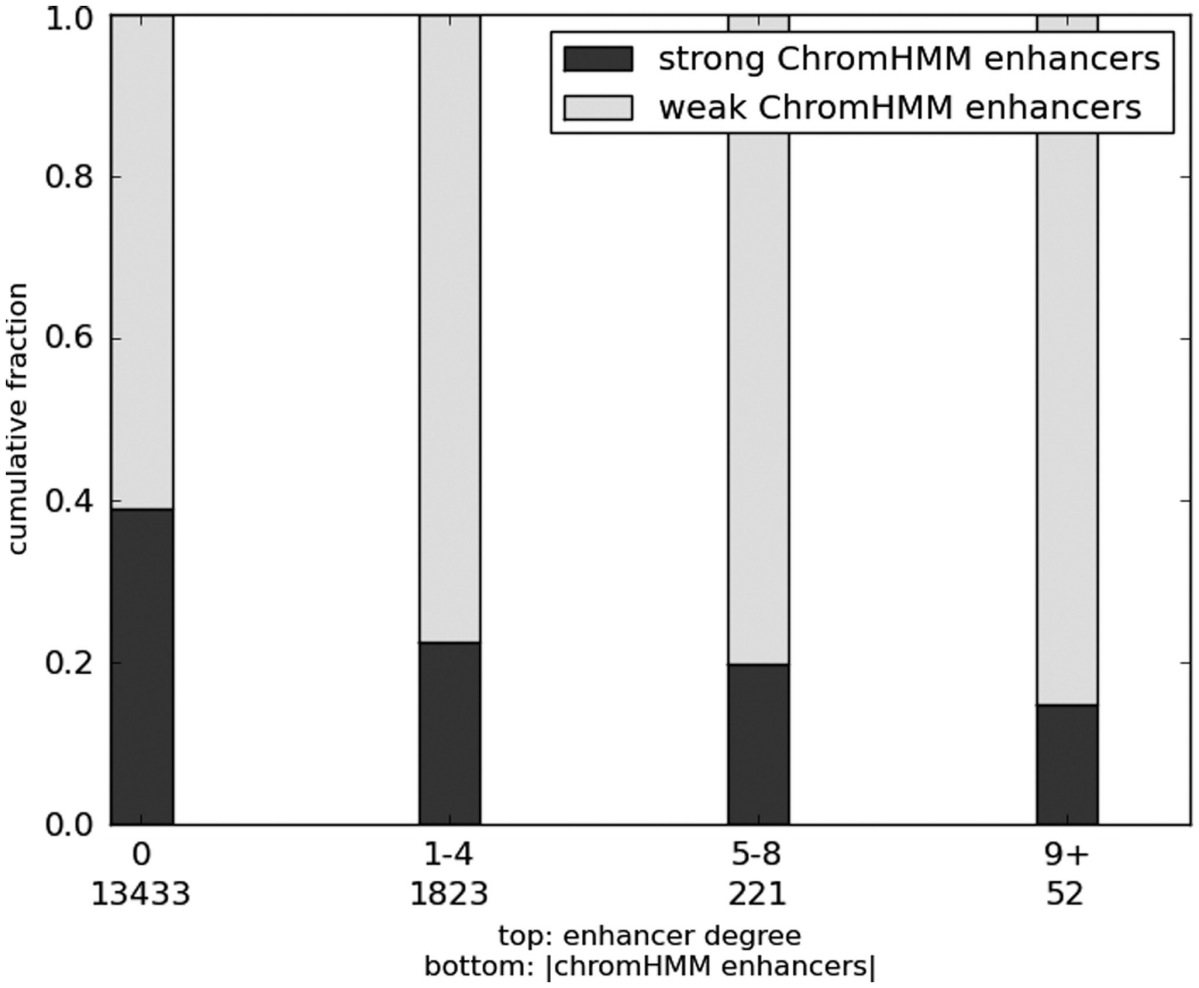
Relative to strong enhancers, weak enhancers are more likely to be coordinately activated with other enhancers. Bar plot shows the relative fractions of all enhancers that are non-ambiguously classified in chromHMM data base as ‘weak’ or ‘strong’ enhancers partitioned into four groups, based on their degree, i.e. the number of other enhancers with which they are epigenetically highly correlated (FDR 0.0001), which is recorded along top row of *x*-axis. Numbers on bottom row indicate the total number of non-ambiguously classified chromHMM enhancers in that bin. The determination of whether an enhancer has 0 neighbors was made at a more relaxed FDR 0.05.

Previous studies have shown that low affinity binding sites for individual TFs tend to cluster on the genome (26), and such clustering of binding sites in regulatory regions has been suggested to cooperate to promote overall functionality via multiple mechanisms (27–30). Extending this notion to the level of enhancers, we assessed whether weak enhancers have a greater proclivity to cooperate. Ernst and Kellis (31) have previously predicted enhancers in the genome based on histone modification patterns using the ChromHMM tool and further classified the enhancers into ‘strong’ and ‘weak’ based on cell-type-specific expression level of the proximal gene. We calculated each enhancer’s ‘degree’, as the number of other enhancers it is correlated with and partitioned enhancers into five bins based on degrees: 0, 1–4, 5–8, ≥9 (other binning schemes do not affect the conclusion). For each bin, we calculated the fraction of ‘strong’ enhancers out of all enhancers overlapping with a ChromHMM enhancer. Figure 4 shows that weak enhancers tend to have correlated activity with several other enhancers, whereas strong enhancers tend to function in smaller groups. For instance, the percentage of strong enhancers having no correlation partners (44%) is significantly higher than that for the weak enhancers (35%) (Fisher exact test *P* = 1.8e-56). Next, we checked whether strong/weak enhancers preferentially interact with other strong/weak enhancers. Even though strong enhancers have fewer interactions, we found that strong enhancers are twice as likely to be correlated with another strong enhancer than expected by chance (Fisher exact test *P* = 1.6e-7). Similarly, weak enhancers preferentially interact with other weak enhancers (Fisher exact test *P* = 0.0002). The aforementioned results are based on an MI FDR threshold of 0.01, but the trend remains significant at FDR = 0.05. Thus, strong and weak enhancers assort with other strong and weak enhancers, respectively.

### Potential roles of TFs and CMEs in correlated enhancer activity

**Figure 5.**
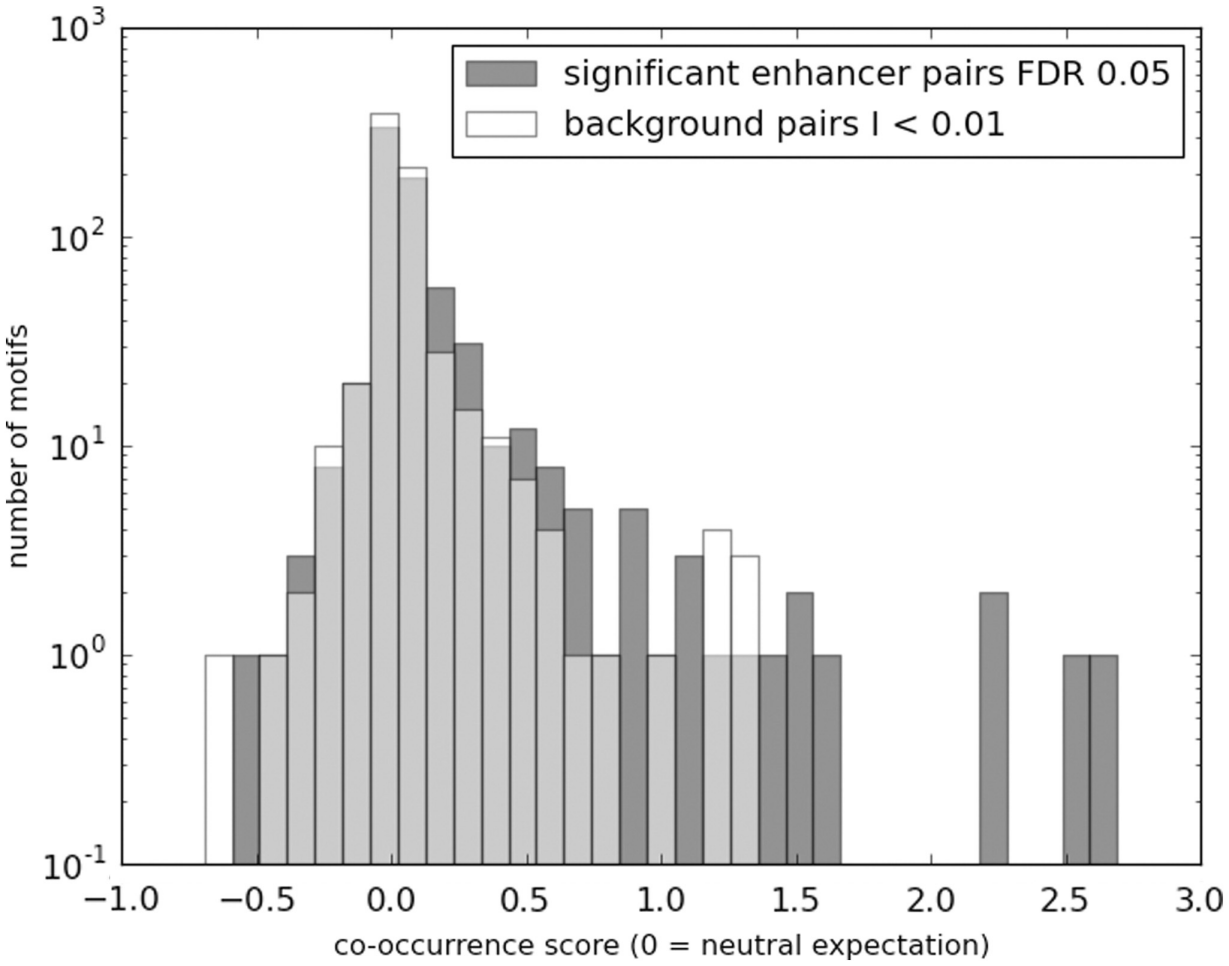
Motif co-occurrence is greater among correlated enhancers relative to background non-correlated enhancer pairs. Histogram shows the log enrichment of motif co-occurrence above random expectation for significantly correlated enhancer pairs (FDR 0.01) (green) compared with the same for background pairs (red). The *x*-axis shows the log of enrichment values, where 0 denotes random expectation, and more positive scores indicate higher enrichment, whereas negative scores indicate higher depletion. The *y*-axis shows the number of motifs with the indicated level of log enrichment. Background pairs were selected based on MI scores <0.01. The ‘10^−1’^ on the *y*-axis is an artifact of the drawing tool and simply represents 0.

It is possible that correlated activities of enhancers are mediated by common TFs as has been shown widely for promoters of co-expressed genes (11). We therefore tested whether correlated enhancer pairs harbor common TF binding sites. We created two sets of enhancer pairs: the ‘foreground’ included the significantly correlated enhancer pairs at FDR = 5% (conclusions remain the same at other thresholds) in each distance bin. ‘Background’ enhancer pairs were randomly chosen from enhancer pairs in each distance bin with MI <0.01. In this context and in what follows, the term ‘Background’ is used to refer to uncorrelated enhancer pairs as opposed to non-enhancer pairs. Next, we identified high-scoring binding sites in each enhancer for each of the 981 vertebrate motifs (see ‘Materials and Methods’ section) and quantified the tendency of a motif to co-occur in correlated enhancers based on a ‘co-occurrence score’ (see ‘Materials and Methods’ section). We found that the overall co-occurrence score distribution for all motifs was significantly higher in the foreground than the background (Figure 5; Wilcoxon test *P* = 6.7e-18). Next, we estimated the significance of co-occurrence for each motif in the foreground by comparing observed and expected co-occurrence frequency using a Chi-squared test. After controlling for multiple testing, at FDR = 0.05, we found 153 motifs with significant co-occurrence (‘Materials and Methods’ section). An identical analysis of background enhancer pairs yielded only 39 motifs. We further filtered the 153 motifs down to the 62 most significant motifs by directly comparing the co-occurrence *P*-values in the foreground and the background using the nominal FDR approach (25) at 5% FDR. Of the 62, 10 were significant in the background. The remaining 52 motifs (Table 1) were used for further analyses.

**Table 1.** Motifs with significantly greater co-occurrence in correlated enhancers than expected (after filtering—see text)

Motif	Co-occurrence Score	*P*-value	*q*-value	Gene
M00649	9.80E-02	0	1.70E-04	MAZ
M01742	1.20E+00	0	2.10E-04	Zfp206
M00986	3.90E-02	0	3.00E-04	Churchill
M00915	5.40E-01	0	3.80E-04	AP-2
M01028	2.70E+00	0	4.30E-04	NRSF
M01783	6.30E-01	0	4.70E-04	SP2
M00431	1.30E-01	0	5.10E-04	E2F-1
M00008	3.30E-01	0	5.60E-04	Sp1
M01199	6.90E-01	0	6.00E-04	RNF96
M01219	4.60E-01	0	6.40E-04	SP1:SP3
M00925	5.40E-02	0	7.30E-04	AP-1
M01253	7.50E-01	0	8.10E-04	CNOT3
M00189	6.80E-01	0	9.00E-04	AP-2
M00255	3.70E-01	0	9.40E-04	GC_box
M01482	2.60E+00	0	9.80E-04	Nkx3-2
M00716	8.20E-01	0	1.00E-03	ZF5
M01267	6.40E-02	0	1.10E-03	FRA1
M00199	9.20E-02	0	1.10E-03	AP-1
M00196	6.30E-01	0	1.20E-03	Sp1
M00800	8.00E-01	0	1.20E-03	AP-2
M00807	3.20E-01	0	1.30E-03	Egr
M00931	4.80E-01	0	1.30E-03	Sp1
M00933	3.20E-01	0	1.40E-03	Sp1
M00932	5.90E-01	0	1.40E-03	Sp1
M00615	1.90E+00	0	1.50E-03	c-Myc:Max
M01303	3.10E-01	0	1.50E-03	SP1
M01588	2.90E-01	0	1.50E-03	GKLF_(KLF4)
M00322	4.30E-01	0	1.60E-03	c-Myc:Max
M00976	2.20E-01	0	1.60E-03	AhR,_Arnt,_ HIF-1
M00720	7.80E-02	0	1.70E-03	CAC-binding_ protein
M01273	4.50E-01	0	1.70E-03	SP4
M01837	1.70E-01	0	1.80E-03	FKLF
M00174	1.10E-01	1.10E-16	1.90E-03	AP-1
M00926	3.80E-02	4.40E-16	1.90E-03	AP-1
M00428	4.60E-02	6.70E-16	2.00E-03	E2F-1
M01593	9.50E-01	1.20E-15	2.10E-03	Zfx
M01104	4.60E-02	2.20E-14	2.10E-03	MOVO-B
M01177	3.20E-01	1.50E-11	2.10E-03	SREBP2
M01230	2.40E-02	1.60E-11	2.20E-03	ZNF333
M01816	1.30E-01	5.60E-11	2.20E-03	ZBP89
M00940	5.50E-01	4.10E-10	2.30E-03	E2F-1
M01597	2.20E-01	9.70E-10	2.30E-03	Zfp281
M01045	3.90E-01	2.70E-09	2.40E-03	AP-2alphaA
M01162	3.00E-02	1.20E-08	2.40E-03	OG-2
M01292	2.00E-02	1.50E-08	2.40E-03	HOXA13
M00378	9.90E-02	1.30E-07	2.50E-03	Pax-4
M00982	6.80E-01	2.00E-07	2.60E-03	KROX
M00644	3.30E-02	3.70E-07	2.60E-03	LBP-1
M01714	3.50E-01	4.70E-07	2.70E-03	KLF15
M01275	2.40E-02	9.80E-07	2.70E-03	IPF1
M01318	1.40E+00	1.60E-06	2.70E-03	Irx-3
M00175	4.70E-02	1.90E-06	2.80E-03	AP-4

Column 1: TRANSFAC Motif ID, Column 2: Co-occurrence score (see text), Column 3: *P*-value, Column 4: Multiple testing corrected *q*-value, Column 5: TF name.

When we repeated the aforementioned analysis by only considering the motifs whose corresponding TF genes are present in tissues where enhancer pair is active, our conclusions are further strengthened (Supplementary Note S1). This also proved true when the aforementioned pair-wise co-occurrence analysis was extended to clusters of correlated enhancers (Supplementary Note S2 and Supplementary Figure S3). Finally, in addition to determining that co-occurring motifs are present more often than expected in correlated enhancer pairs, we also observe that correlated enhancer pairs share overall greater numbers of motifs than expected (Supplementary Note S3). Taken together, the aforementioned analyses suggest that epigenetically correlated enhancers share TF binding motifs significantly more frequently than expected suggesting a role of TFs in enhancer co-regulation.

Next, we assessed, using machine learning, whether the presence of common motifs can predict correlated activity of a pair of enhancers (see Supplementary Note S4 for details). To summarize, using 10-fold cross-validation, a support vector machine based on 981 motif matches, as feature set was able to discriminate between the foreground and the background enhancer pairs with an overall average classification accuracy of 73%. Importantly, there was little reduction in performance (70%) when the model used only the 52 significantly co-occurring motifs detected earlier in the text.

Finally, we probed the potential involvement of CME in regulating correlated enhancer activities. We assessed each of the 828 CMEs for preferential interaction with significant motifs relative to the other motif, using a Fisher Exact test (see Supplementary Note S5 for details). At FDR = 5%, we detected 28 CMEs to preferentially interact with significant motifs (Table 2). In contrast, there was no CME that preferentially interacted with non-significant motifs. Although this result should be interpreted with caution owing to innately noisy PPI data, the analysis nonetheless reveals a small set of CMEs that preferentially interact with co-occurring motifs.

**Table 2.** CME that preferentially interact with significantly co-occurring motifs (Table 1)

CME	*P*-value	Interaction frequency	Description
ENSP00000336750	5.50E-04	5.50%	Suppressor of Ty 7 (*Saccharomyces cerevisiae*)-like
ENSP00000308227	5.90E-04	9.60%	High mobility group AT-hook
ENSP00000264709	9.60E-04	8.20%	DNA (cytosine-5-)-methyltransferase 3 alpha
ENSP00000362649	1.20E-03	16.00%	Histone deacetylase 1
ENSP00000231509	1.60E-03	12.00%	Nuclear receptor subfamily ‘3’, group ‘C’, member 1
ENSP00000349508	2.30E-03	6.80%	Chromodomain helicase DNA-binding protein 4
ENSP00000278823	2.40E-03	6.20%	Metastasis associated 1 ‘family’, member 2
ENSP00000367207	2.90E-03	15.00%	v-myc myelocytomatosis viral oncogene homolog (avian)
ENSP00000343325	2.90E-03	5.50%	Protein kinase N1
ENSP00000263119	4.20E-03	6.20%	Calcineurin-binding protein 1
ENSP00000362674	5.30E-03	5.50%	Histone deacetylase 8
ENSP00000334061	5.40E-03	6.20%	Histone deacetylase 6
ENSP00000386759	7.30E-03	6.80%	SET domain containing 2
ENSP00000302967	9.20E-03	10.00%	Histone deacetylase 3
ENSP00000352516	9.50E-03	8.20%	DNA (cytosine-5-)-methyltransferase 1
ENSP00000284384	1.20E-02	6.80%	Protein kinase ‘C’, alpha
ENSP00000349049	1.30E-02	5.50%	Lysine (K)-specific demethylase 1A
ENSP00000225983	1.40E-02	8.20%	Histone deacetylase 5
ENSP00000381331	1.50E-02	9.60%	Histone deacetylase 2
ENSP00000371067	2.30E-02	8.20%	Janus kinase 2
ENSP00000264606	2.40E-02	7.50%	Histone deacetylase 4
ENSP00000264010	2.50E-02	6.20%	CCCTC-binding factor (zinc finger protein)
ENSP00000268712	2.50E-02	9.60%	Nuclear receptor corepressor 1
ENSP00000337088	2.70E-02	6.20%	Multiple endocrine neoplasia I
ENSP00000356480	2.80E-02	5.50%	Ring finger protein 2
ENSP00000231487	2.90E-02	6.20%	S-phase kinase-associated protein 1
ENSP00000263253	3.00E-02	15.00%	E1A-binding protein p300
ENSP00000267163	3.10E-02	9.60%	Retinoblastoma 1

Column 3 denotes the percentage of significant motifs interacting with the CME.

### Correlated enhancers are spatially proximal

We expect the correlated activity of non-proximal enhancers to be associated with their spatial proximity in the nucleus. We estimated the fraction of correlated enhancer pairs that are spatially proximal based on Hi-C data (GSE18199) (32). We note that the Hi-C data was obtained from human K562 and HIC_gm06690 cell lines, whereas DHS correlation was obtained across 37 primary cell types. It is known that spatially interacting regions are enriched for DHS (33). We controlled for this by ensuring that in each distance bin, the background enhancer pairs were selected such that their average pair-mean DHS across cell types was within 2% of the corresponding average for foreground pairs. We compared foreground and background enhancer pairs in terms of the fraction of pairs that are spatially proximal according to the K562 Hi-C experiment, using Fisher Exact Test. We found that overall, the foreground enhancer pairs showed a greater coincidence with Hi-C data (*P* = 0.01). Even when we include only the top 10% most confident Hi-C pairs, the *P* = 0.03. When we repeat the aforementioned tests using the HIC_gm06690 Hi-C data, the corresponding *P*-values are 0.02 and 0.009. These results suggest that spatial proximity of the chromosomal regions is associated, albeit weakly, with correlated enhancer activities. The weak association may be due to cell-type specificity of spatial proximity (see ‘Discussion’ section).

### Genes near correlated enhancers have correlated expression and shared function

We hypothesized that the gene targets of highly correlated enhancers are themselves correlated in their expression. Although the targets of enhancers are largely unknown, as a first approximation, we mapped each enhancer to its nearest gene as a putative target (34). For each gene, we obtained from GEO (20) the normalized RNA-seq transcript counts from 15 of the 72 tissue types and calculated the Spearman correlation between vectors of transcript counts. For the foreground enhancer pairs at FDR 1% (results are comparable for other FDR thresholds), we found that the median Spearman correlation of expression of the target genes was 0.31, whereas for the background, it was only 0.18 (Wilcoxon rank-sum test *P* = 2.1e-74). It indicates that epigenetically correlated enhancers tend to have co-expressed target genes.

Our analyses thus far suggest that correlated enhancer pairs have (i) a greater motif co-occurrence (section ‘Potential roles of TFs and CMEs in correlated enhancer activity’) and (ii) greater co-expression between their target genes (section ‘Genes near correlated enhancers have correlated expression and shared function’). Therefore, we assessed directly whether motif co-occurrence in enhancers is predictive of correlated expression in their target genes, regardless of correlated activity of the enhancers. Ten thousand enhancer pairs were sampled without regard for their correlation. The Jaccard index for motif sharing between enhancers and gene co-expression for putative target genes was estimated as aforementioned. Based on linear regression of expression correlation against the corresponding enhancer pairs’ Jaccard indices, we found the two to be highly positively associated with a slope of 0.26 (*P* = 4.4e-26 for null hypothesis that slope = 0), suggesting that shared motifs in enhancers is predictive of their target genes’ co-expression.

Next, we tested whether targets of correlated enhancers are functionally related. For each enhancer pair, we checked whether target genes, if they are different, share a Gene Ontology (GO) biological process. We only considered specific GO terms including at most 200 genes (this threshold was varied from 200 to 2000). We found that the foreground enhancer pairs consistently share a GO term more frequently than the background; the difference between them varying between 11 and 30%. This difference is significant (Fisher Exact test *P* < 0.05) for all but one thresholds where it was marginally significant with *P* = 0.06. This suggests that gene targets of correlated enhancer pairs tend to be functionally related.

### Targets of correlated enhancer clusters have correlated expression and shared function

We extended our analyses previous sections to ‘clusters’ of correlated enhancers. We identified enhancer clusters for a variety of parameters (Supplementary Note S6) pertaining to cluster size, intra-cluster MI and fraction of enhancers sharing a motif. For each enhancer cluster, a control cluster was created from non-correlated enhancers that mirrored the former’s size and genomic footprint (i.e. intra-cluster genomic distances). We found that putative targets of correlated clusters (i.e. the set of genes nearest to each enhancer) were more highly correlated to each other in their RNA-seq transcript counts across 15 cell types than were background clusters. For the entire range of parameters, mean expression correlation within foreground clusters was consistently greater than that for background clusters. Owing to the variability in cluster counts for different parameters, *P*-values ranged from 0.02 to 4.1e-15 (Wilcoxon rank-sum test). These results suggest that gene targets of correlated enhancer clusters with shared motifs are co-expressed and presumably co-regulated.

Next, we assessed enrichment of GO biological processes amongst the targets of an enhancer cluster using R’s GOstats package. Enhancer clusters also revealed consistently greater GO functional enrichment than the background clusters. Across all parameter settings, the ratio of number of enriched GO terms (at FDR 0.01) per cluster was on average 3-fold higher in the foreground (19.1 terms per cluster). As an example, for the parameter setting with the greatest fold enrichment of GO terms, the terms are shown in Supplementary Table S2. These terms are consistently revealed across all parameters settings. Together, the GO enrichment and gene expression results suggest that co-expression of genes with shared function is coordinately regulated across tissues by enhancers that share motifs and are epigenetically correlated across the same tissues.

### Concordant cell type specificity of enhancer clusters and their target genes

Enhancers are believed to regulate cell-type-specific gene expression. We tested whether there is cell-specificity among the gene targets of correlated enhancers. For identifying cell-type-specificity of gene expression, we used the online tool CTen (35), which compares input genes with a database of highly expressed cell-specific genes found in public microarray databases and reports any significant overlaps. Enhancer clusters and associated target genes were identified with three parameter settings resulting in 42, 122 and 182 clusters, with average cluster sizes 64, 31 and 19 genes, respectively. Background gene sets were obtained as in previous section. Our results indicated high tissue enrichment in the gene targets of correlated enhancer clusters. For instance, with 42 clusters, we found enrichment (FDR = 1%) for 23 tissue-specific gene sets involving 16 clusters, whereas no enrichment was detected in the corresponding background clusters; results are qualitatively similar for other parameter settings.

Next, we hypothesized that if the genes targeted by an enhancer cluster are expressed in specific cell types, then the enhancers in the cluster should have high DHS in the same cell type(s). We determined the average DHS of an enhancer cluster in ENCODE cell types and obtained the DHS-based rank of the cell type in which the corresponding gene cluster was specifically expressed according to CTen; mapping between CTen tissue types and ENCODE cell types was manually determined and organized into classes (Supplementary Table S3). For a clustering parameter, we obtained the median rank for the resulting enhancer clusters as well as median rank for an equivalent set of background clusters. We found that across eight different clusterings, the median ranks of enhancer clusters ranged from 4 to 8 with a mean of 6, whereas the expected median rank is 11.5. Overall, this result suggests that there is concordance between enhancer clusters and targeted gene clusters in their tissue-specific activity.

**Figure 6.**
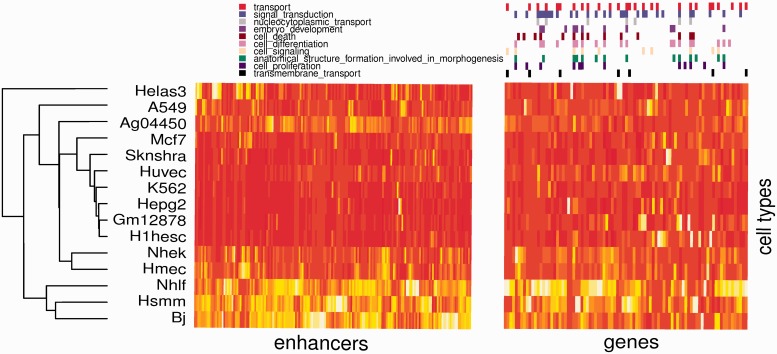
Tissue activity profile of an enhancer cluster and the corresponding target genes. Left Panel: Tissue-specific DHS activity for 179 coordinately activated enhancers. Data are shown only for 15 cell types for which RNA-seq data was also available. Rows (cell types) and columns (enhancers) are hierarchically clustered. Right Panel: Corresponding expression of the 98 target genes in the same 15 cell types. Gene membership in GO slim categories that are highly enriched is displayed above the heat plot. Columns (genes) have been clustered independently, however, row order is preserved from the enhancer heatmap. In both maps, deeper shades of color indicate higher values.

Figure 6 shows an illustrative example of an enhancer cluster (179 enhancers) and corresponding gene cluster (98 genes) with tissue-specific activities across 15 cell types. The DHS profiles of the enhancers (Figure 6, left panel) mirror the expression profiles of the genes (Figure 6, right panel). These genes are highly expressed in a number of cancer cell lines and an embryonic stem cell line, combined with markedly lower expression in normal adult somatic cells and are highly enriched for terms related to intra- and inter-cellular signal processing, and regulation of transcription (Supplementary Table S4).

## DISCUSSION

Based on a systematic analysis of correlated enhancer activities across 72 cell types, we found a broad range of evidence that support coordinated enhancer activities, potentially mediated by TFs, CMEs and spatial chromatin structure. Our analyses are based on stringent controls at various stages to maximize the robustness of our conclusions. First, we explicitly control for observed autocorrelation along the genome in DHS levels, which would otherwise inappropriately make neighboring enhancers seem correlated. Second, when appropriate, we remove transitive correlations between enhancers. Third, when analyzing a group of enhancer pairs, we create an appropriate negative control by selecting uncorrelated enhancer pairs with similar inter-enhancer distances. Fourth, to control for cell type similarities, 37 representative cell types were selected from 72 cell types. Fifth, significantly co-occurring motifs in enhancer pairs were screened for high likelihood of active tissue-specific TF binding. Sixth, dependencies owing to motif similarity were addressed by clustering motifs. Seventh, clustering parameters settings that included cutoff for MI, minimum size and minimum level of motif enrichment were varied to ensure robustness of pattern discovery at the network level. For individual analyses, additional controls were used to ensure robustness of our conclusions.

P300 binding has been shown to be an accurate marker of tissue relevant enhancers (5). The base set of 98 000 enhancers was identified based on P300 binding in one of the four cell types. P300 binding is a reasonable marker of candidate enhancer for the intended aim of our work, namely, to investigate coordinated enhancer activities and test hypotheses concerning its functional underpinning and consequences. Although there are alternative ways of identifying the candidate enhancers, such as ChromHMM (31), the combination of DHS and 5C (34), and other epigenomic marks (7), they all can have false positives. Moreover, using DHS as a proxy for an enhancer’s tissue-specific activity allowed us to take advantage of the many tissues for which DHS data are currently available, without introducing circular dependence. Even though individual enhancers may be false positives, we infer correlated activity based on highly significant DHS correlation across 37 independent cell types after controlling for potential autocorrelation. Despite noise at the level of individual enhancers, we observe significant patterns when comparing enhancers with coordinated activities with background enhancer pairs, which notably are derived from the same set of enhancers. Approximately 53% of our enhancers overlap with those predicted by ChromHMM. To further ensure the robustness of our conclusions, we repeated some of our analyses separately on the subset of enhancers supported by chromHMM and the ones not predicted by ChromHMM. In both disjoint data sets, we still observed that correlated enhancers had significant motif co-occurrence, and that the potential targets of correlated enhancers were significantly correlated in their expression and function.

The goal of identifying the full complement of enhancers that drive transcriptional regulation in a specific context remains largely unmet. This work suggests a useful paradigm for organizing enhancers into clusters of coordinated activities. These clusters of enhancers, given their high cross-tissue concordance in epigenetic state, are likely to participate in coordinate transcription regulation of specific genes, or more likely, pathways. Presently, researchers treat enhancers and their gene targets predominantly as independent edges in a graph. By leveraging prior knowledge of these clusters, searches for enhancer-target genes will benefit from both greater sensitivity and greater specificity.

In addition to finding clusters of enhancers ostensibly involved in coordinate regulation of gene transcription, we also examined the nature of the clusters. We asked, for example, whether there was a pattern in clusters with regard to enhancer strength, as manifested in the expression level of target genes. We found that strong enhancers are much more likely to function in isolation than are weak enhancers. Moreover, strong and weak enhancers assort with enhancers of the same kind: strong (weak) enhancers prefer to interact with strong (weak) enhancers.

TF binding motifs can exert influence on enhancer activity. We found that shared motifs can predict correlated activities of a pair of enhancers. Even though there is no qualitative difference in density and composition of motifs between enhancers that are involved in coordinate regulation and enhancers that are not, certain motifs preferentially co-occur in correlated enhancers. This could be explained if enhancers with shared motifs respond in unison to a common modulator, such as an allosterically regulated TF, or a pioneer TF that can interact with and recruit CMEs. Indeed, we found that co-occurring motifs do preferentially interact with a subset of CMEs.

We found that correlated enhancers that are in genomic proximity share fewer significantly co-occurring motifs relative to those that are far apart (Table 3). This, in conjunction with a greater propensity for coordinated activity for nearby enhancers (Figure 3), suggests alternative mechanisms for proximal and distal enhancer pairs’ coordinated activities. Greater motif sharing between distant enhancer pairs is consistent with a more active role of motifs in establishing coordinated activity, with or without influencing spatial proximity.

**Table 3.** Motif sharing between coordinated enhancer pairs and the background

Max dist between enhancers (kB)	Correlated enhancer pairs (FDR 0.0001)		Background enhancer pairs (I < 0.01)	
	Mean Jaccard (all motifs)^a^	Median Jaccard (all motifs)^a^	Mean Jaccard (all motifs)^a^	Median Jaccard (all motifs)^a^
20	0.32	0.32	0.3	0.3
200	0.32	0.32	0.29	0.28
1000	0.31	0.31	0.29	0.29
20 000	0.31	0.31	0.28	0.28
Overall	0.31	0.31	0.29	0.29
	Mean Jaccard (significant motifs)^b^	Median Jaccard (significant motifs)^b^	Mean Jaccard (significant motifs)^b^	Median Jaccard (significant motifs)^b^
20	0.22	0.14	0.12	0
200	0.28	0.2	0.11	0
1000	0.29	0.2	0.11	0
20 000	0.3	0.25	0.11	0
Overall	0.28	0.2	0.11	0

^a^This table shows results of Wilcoxon rank-sum tests comparing the extent of motif overlap in correlated enhancer pairs (FDR 0.0001) to that in background pairs, with one test per distance bin. All 981 vertebrate motifs in the TRANSFAC database were used.

^b^Same as (a), except that overlap is evaluated only for the significantly co-occurring motifs in correlated enhancers.

Overall, our analysis suggests that mirroring the known organization of genes into functionally linked co-expressed modules, distal enhancers regulating such genes are also organized into modules of correlated activity across cell types. Strong and weak enhancers exhibit differential correlated activity and assortativity with strong and weak enhancers, respectively. The observed organization of mammalian enhancers into correlated networks is likely mediated by the joint action of TFs through shared motifs, CMEs and spatial chromatin structure.

## SUPPLEMENTARY DATA

Supplementary Data are available at NAR Online: Supplementary Tables 1–4, Supplementary Figures 1–3 and Supplementary Notes 1–6.

## FUNDING

Funding for open access charge: National Institutes of Health [R01GM100335 to S.H.].

*Conflict of interest statement.* None declared.

## Supplementary Material

Supplementary Data
