# Supplementary material for: Enhancer networks revealed by correlated DNAse hypersensitivity states of enhancers
Source: Nucleic Acids Res. 2013 May 21;41(14):6828–38. doi: 10.1093/nar/gkt374 (PMC3737527; doi:10.1093/nar/gkt374)
Supplement: Supplementary Data [file supp_41_14_6828__index.html]

Enhancer networks revealed by correlated DNAse hypersensitivity states of enhancers — Enhancer networks revealed by correlated DNAse hypersensitivity states of enhancers — Supplementary Data 

# Enhancer networks revealed by correlated DNAse hypersensitivity states of enhancers

## Supplementary Data

files

**Files in this Data Supplement:**

- Supplementary Data - pdf file
