# Supplementary material for: Enhancer networks revealed by correlated DNAse hypersensitivity states of enhancers
Source: Nucleic Acids Res. 2013 May 21;41(14):6828–38. doi: 10.1093/nar/gkt374 (PMC3737527; doi:10.1093/nar/gkt374)
Supplement: Supplementary Data [file supp_gkt374_nar-00172-x-2013-File002.pdf]

Supplemental Figures

Figure 1. Histogram shows the distribution of the number of tissues (x-axis) a given enhancer is active in (out of 37 tissues possible).

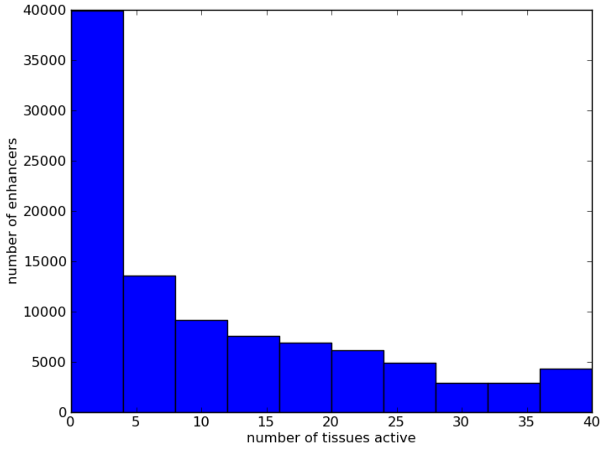

Figure 2. Fraction of significantly correlated enhancer pairs decreases monotonically with increasing distance between the enhancers, even when *a)* an FDR test conducted on a common pooled background is used. (Bin-wise fractions reflect post-test partitioning of enhancer pairs based on genomic distance), and *b)* a background of trans-chromosomal pairs is used.

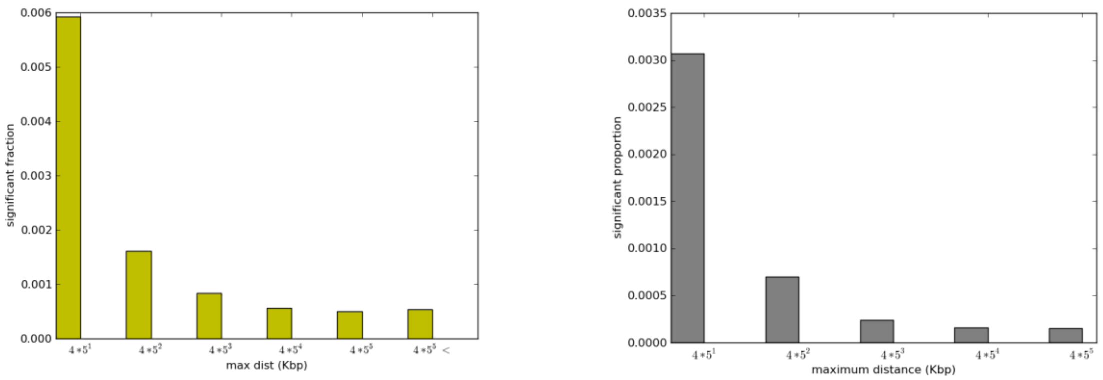

(a)

(b)

Figure 3. Illustrative example of an enhancer cluster. The figure shows on the genome browser a representative cluster of enhancers comprising 117 enhancer spread throughout chromosome 2. This cluster includes 12 strong (blue ticks) and 54 weak enhancers (red ticks) as annotated by ChromHMM. DHS (black ticks) in 5 representative cell types are shown for all enhancers. The figure clearly illustrates the correlated activity of these enhancers across the cell types. This cluster, which was constructed without regard to motif co-occurrence, in fact also broadly shares 2 motifs (magenta ticks).

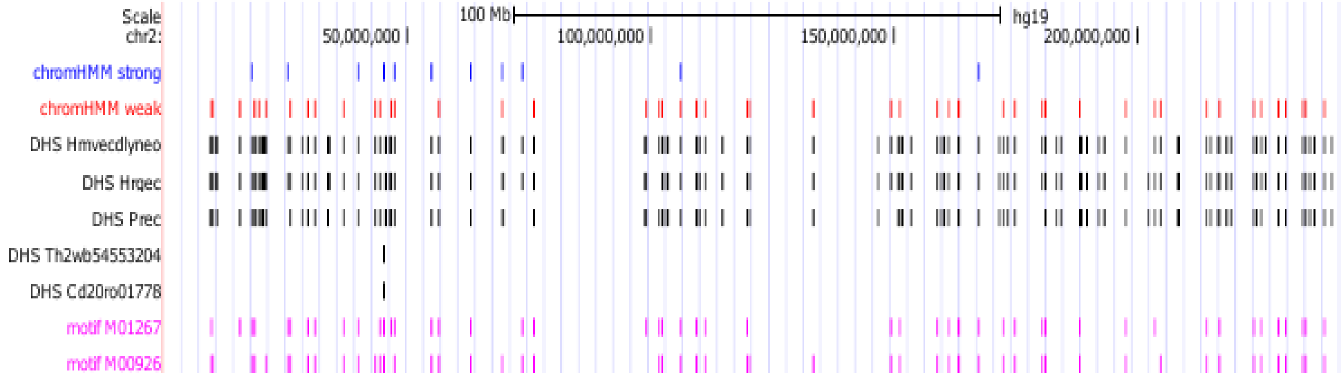

Supplemental Tables

Supplementary Table 1. 73 cell types were sorted into 37 clusters. One cell type from each cluster (first in row) was used as the representative for the cluster. See text for how the representative was selected.

Cluster	Cell Types	
1	A549	
2	Aoaf	M059j
3	Be2c	
4	Cd20ro01778	
5	Gm04503	
6	Gm04504	
7	Hah	
8	Hasp	Nt2d1
9	Hbmec	Hff
10	Hipe	
11	Hmf	
12	Hmvecdad	
13	Hmvecdblneo	
14	Hmvecdlyneo	
15	Hmveclly	
16	Hpaf	Hsmmt
17	Hrgec	Th1wb54553204
18	Hs5	
19	Hsmm	

20	Huvec Hbvp Hct116 Hmec Hmvecdblad Hmvecdneo Hpdlf Nhek Rpmi7951 Th1 Tregwb83319432 Wi38
21	Jurkat
22	Mcf7 Lhcnm2
23	Monocd14ro1746
24	Msc
25	Nha
26	Nhbera
27	Nhdfad Hmveclbl Hpaec
28	Prec
29	Rptec Gm12864 Hac Hcfaa Hconf Th17
30	Sknmc
31	T47d Cd34mobilized
32	Th1wb33676984
33	Th2
34	Th2wb33676984
35	Th2wb54553204 Cd4naivewb78495824
36	Tregwb78495824 Cd4naivewb11970640 H7es Hbvsmc Hffmyc Hmvecdlyad Hpf Hs27a Hvmf Lncap
37	Werirb1

Supplementary Table 2. Gene Ontology (GO) annotation terms for the clusters of target genes corresponding to correlated enhancer clustering with the highest ratio of enrichment terms between itself and a background gene cluster. In this list are GO terms separated by targeted gene cluster with adjusted p-values < 0.0005 and that are supported by three or more genes in the cluster. 7 of 52 clusters were enriched for at least one term that met this highly stringent standard. There were 149 separate instances of enrichment. This enhancer cluster was identified using the following parameters: min mean mutual information = 0.2, minimum cluster size = 20, minimum percent occupancy for most enriched motif = 0.0. Background clusters are matched for chromosome, the number of enhancers and signature of inter-enhancer distances, but consist of otherwise random enhancers. GO enrichment analysis performed with R's GOstats package. Adjusted p-value = 0.05*p-value/ q-value.

---

cluster size: 65 genes

**Enriched term**	**#genes**	**Adjusted p-value**	**Description**
GO:0009790	4	3.3e-04	embryo development
GO:0007411	3	3.3e-04	axon guidance
GO:0051179	10	3.3e-04	localization
GO:0009605	5	3.3e-04	response to external stimulus
GO:0051093	3	3.5e-04	negative regulation of developmental process
GO:0048519	8	3.5e-04	negative regulation of biological process
GO:0045597	3	3.5e-04	positive regulation of cell differentiation
GO:0016337	3	3.5e-04	cell-cell adhesion
GO:0001775	4	3.5e-04	cell activation
GO:0060284	3	3.6e-04	regulation of cell development

GO:0051960	3	4.0e-04	regulation of nervous system development
GO:0048523	8	4.2e-04	negative regulation of cellular process
GO:0065008	8	4.2e-04	regulation of biological quality
GO:0072358	4	4.2e-04	cardiovascular system development
GO:0072359	4	4.2e-04	circulatory system development
GO:0045596	3	4.2e-04	negative regulation of cell differentiation
GO:0051129	3	4.3e-04	negative regulation of cellular component organization
GO:0048568	3	4.3e-04	embryonic organ development
GO:0071845	3	4.3e-04	cellular component disassembly at cellular level
GO:0051239	6	4.3e-04	regulation of multicellular organismal process
GO:0022411	3	4.3e-04	cellular component disassembly
GO:0050767	3	4.3e-04	regulation of neurogenesis
GO:0007155	5	4.3e-04	cell adhesion
GO:0022610	5	4.3e-04	biological adhesion
GO:0007507	3	4.3e-04	heart development
GO:0050793	5	4.3e-04	regulation of developmental process
GO:0030182	5	4.5e-04	neuron differentiation
GO:2000026	5	5.0e-04	regulation of multicellular organismal development

---

cluster size: 141 genes

GO:0048812	4	2.3e-04	neuron projection morphogenesis
GO:0048667	4	2.3e-04	cell morphogenesis involved in neuron differentiation
GO:0001525	3	2.3e-04	angiogenesis
GO:0051172	5	2.3e-04	negative regulation of nitrogen compound metabolic process
GO:0048585	4	2.3e-04	negative regulation of response to stimulus
GO:0048568	3	2.3e-04	embryonic organ development
GO:0007409	4	2.3e-04	axonogenesis
GO:0001558	3	2.3e-04	regulation of cell growth
GO:0051090	3	2.3e-04	regulation of transcription factor activity
GO:0048468	6	2.3e-04	cell development
GO:0002009	3	2.3e-04	morphogenesis of an epithelium
GO:0050767	3	2.3e-04	regulation of neurogenesis
GO:0090046	3	2.3e-04	regulation of transcription regulator activity
GO:0010629	5	2.3e-04	negative regulation of gene expression
GO:0007507	3	2.3e-04	heart development
GO:0007399	7	2.3e-04	nervous system development
GO:0045934	5	2.3e-04	negative regulation of nucleobase, nucleoside, nucleotide and nucleic acid metabolic process
GO:0016481	5	2.4e-04	negative regulation of transcription
GO:0032501	16	2.5e-04	multicellular organismal process
GO:0001503	3	2.7e-04	ossification
GO:0035239	3	2.7e-04	tube morphogenesis
GO:0006357	6	2.8e-04	regulation of transcription from RNA polymerase II promoter

GO:0042127	6	2.9e-04	regulation of cell proliferation
GO:0032582	3	3.0e-04	negative regulation of gene-specific transcription
GO:0008283	7	3.0e-04	cell proliferation
GO:0050673	3	3.3e-04	epithelial cell proliferation
GO:0009887	5	3.3e-04	organ morphogenesis
GO:0042692	3	3.3e-04	muscle cell differentiation
GO:0007411	4	3.7e-04	axon guidance
GO:0009890	6	3.7e-04	negative regulation of biosynthetic process
GO:0002697	3	3.8e-04	regulation of immune effector process
GO:0048869	10	3.9e-04	cellular developmental process
GO:0031327	6	3.9e-04	negative regulation of cellular biosynthetic process
GO:0010553	3	3.9e-04	negative regulation of gene-specific transcription from RNA polymerase II promoter
GO:0009892	7	4.1e-04	negative regulation of metabolic process
GO:0031324	7	4.2e-04	negative regulation of cellular metabolic process
GO:0035295	4	4.2e-04	tube development
GO:0010605	7	4.2e-04	negative regulation of macromolecule metabolic process
GO:0045892	5	4.2e-04	negative regulation of transcription, DNA-dependent
GO:0051253	5	4.2e-04	negative regulation of RNA metabolic process
GO:0009605	7	4.2e-04	response to external stimulus
GO:2000113	6	4.2e-04	negative regulation of cellular macromolecule biosynthetic process
GO:0022603	4	4.2e-04	regulation of anatomical structure morphogenesis
GO:0060284	4	4.2e-04	regulation of cell development
GO:0001763	3	4.2e-04	morphogenesis of a branching structure
GO:0019216	3	4.2e-04	regulation of lipid metabolic process
GO:0030154	10	4.2e-04	cell differentiation
GO:0050678	3	4.2e-04	regulation of epithelial cell proliferation
GO:0031347	4	4.2e-04	regulation of defense response
GO:0006935	5	4.2e-04	chemotaxis
GO:0042330	5	4.2e-04	taxis
GO:0072358	5	4.2e-04	cardiovascular system development
GO:0072359	5	4.2e-04	circulatory system development
GO:0061061	4	4.2e-04	muscle structure development
GO:0010558	6	4.2e-04	negative regulation of macromolecule biosynthetic process
GO:0061138	3	4.2e-04	morphogenesis of a branching epithelium
GO:0050727	3	4.9e-04	regulation of inflammatory response
GO:0051146	3	4.9e-04	striated muscle cell differentiation
GO:0048754	3	5.0e-04	branching morphogenesis of a tube

---

cluster size: 33 genes

GO:0006936	3	0	muscle contraction
GO:0051259	3	0	protein oligomerization
GO:0003012	3	0	muscle system process
GO:0003013	3	0	circulatory system process

GO:0008015	3	0	blood circulation
GO:0061061	3	0	muscle structure development
GO:0035556	6	0	intracellular signal transduction
GO:0022607	5	0	cellular component assembly
GO:0010627	3	0	regulation of intracellular protein kinase cascade
GO:0050794	13	0	regulation of cellular process
GO:0044085	5	0	cellular component biogenesis

---

cluster size: 6 genes

GO:0007268	4	0	synaptic transmission
GO:0019226	4	0	transmission of nerve impulse
GO:0035637	4	0	multicellular organismal signaling

---

cluster size: 27 genes

GO:0001775	5	0	cell activation
GO:0001568	4	0	blood vessel development
GO:0001944	4	0	vasculature development
GO:0051716	11	0	cellular response to stimulus
GO:0007265	3	0	Ras protein signal transduction
GO:0007166	7	0	cell surface receptor linked signaling pathway
GO:0072358	4	0	cardiovascular system development
GO:0072359	4	0	circulatory system development
GO:0007165	9	0	signal transduction
GO:0006928	4	0	cellular component movement
GO:0007167	4	0	enzyme linked receptor protein signaling pathway
GO:0023052	9	0	signaling

---

cluster size: 53 genes

GO:0045785	3	0	positive regulation of cell adhesion
GO:0007167	6	0	enzyme linked receptor protein signaling pathway
GO:0071844	6	0	cellular component assembly at cellular level
GO:0040007	5	0	growth
GO:0030155	3	0	regulation of cell adhesion
GO:0032268	6	0	regulation of cellular protein metabolic process
GO:0051246	6	0	regulation of protein metabolic process
GO:0048589	3	0	developmental growth
GO:0031399	5	0	regulation of protein modification process
GO:0034622	4	0	cellular macromolecular complex assembly
GO:0071845	3	0	cellular component disassembly at cellular level
GO:0022411	3	0	cellular component disassembly
GO:0009967	4	0	positive regulation of signal transduction

GO:0048584	5	0	positive regulation of response to stimulus
GO:0043623	3	0	cellular protein complex assembly
GO:0022607	6	0	cellular component assembly
GO:0010647	4	0	positive regulation of cell communication
GO:0023056	4	0	positive regulation of signaling
GO:0031401	3	0	positive regulation of protein modification process
GO:0007169	4	0	transmembrane receptor protein tyrosine kinase signaling pathway
GO:0044085	6	0	cellular component biogenesis
GO:0042060	4	0	wound healing
GO:0001932	4	0	regulation of protein phosphorylation

---

clusterID 38 cluster size: 53 genes

GO:0048583	4	3.5e-04	regulation of response to stimulus
GO:0007267	3	3.5e-04	cell-cell signaling
GO:0022008	3	3.6e-04	neurogenesis
GO:0050793	3	3.6e-04	regulation of developmental process
GO:0048731	5	3.7e-04	system development
GO:0048699	3	3.8e-04	generation of neurons
GO:0030182	3	4.0e-04	neuron differentiation
GO:0048518	5	4.0e-04	positive regulation of biological process
GO:0051128	3	4.0e-04	regulation of cellular component organization
GO:2000026	3	4.1e-04	regulation of multicellular organismal development
GO:0030030	3	4.2e-04	cell projection organization
GO:0048522	5	4.4e-04	positive regulation of cellular process
GO:0045595	3	4.8e-04	regulation of cell differentiation
GO:0048666	3	4.9e-04	neuron development

Supplementary Table 3. Mapping of tissues between CTen and ENCODE databases. We clustered the 84 tissue types in the CTen database and the 72 types in the ENCODE DHS database into 34 and 23 cytologically motivated classes, respectively. Agreement in tissue enrichment was assessed based on the 17 classes, shown below, that are shared between CTen and ENCODE.

**Encode cell type (enhancer domain)**	**Tissue class**	***Cten* cell type (gene domain)**
Cd20ro01778	blood	721 b lymphoblasts
Cd34mobilized		bdca4+ dentritic cells
Cd4naivewb11970640		cd19+ b cells
Cd4naivewb78495824		cd33+ myeloid
Gm12864		cd34+
Jurkat		cd4+ t cells
Th1		cd56+ nk cells
Th17		cd71+ early erythroid

Th1wb33676984		cd8+ t cells
Th1wb54553204		leukemia chronic myelogenous k-562
Th2		leukemia lymphoblastic (molt-4)
Th2wb33676984		leukemia promyelocytic hl-60
Th2wb54553204		lymph node
Tregwb78495824		lymphoma burkitts (daudi)
Tregwb83319432		lymphoma burkitts (raji)
		whole blood
Nhbera	bronchial epithelium	bronchial epithelial cells
Hs27a	bone marrow	bone marrow
Hs5		
Be2c	brain	amygdala
Hah		caudate nucleus
M059j		cingulate cortex
Nha		globus pallidus
Sknmc		hypothalamus
		medulla oblongata
		occipital lobe
		olfactory bulb
		parietal lobe
		pineal day
		pineal night
		pituitary
		pons
		prefrontal cortex
		subthalamic nucleus
		temporal lobe
		thalamus
		whole brain
Hac	cerebellum	cerebellum
		cerebellum peduncles
Hct116	colon	colorectal adenocarcinoma
		colon
Aoaf	endothelium	cd105+ endothelium
Hbmec		
Hbvp		
Hbvsmc		
Hmvecdad		
Hmvecdblad		
Hmvecdblneo		
Hmvecdlyad		
Hmvecdlyneo		
Hmvecdneo		
Hmveclbl		
Hmveclly		
Hpaec		
Hpaf		
Huvec		
Hconf	eye	ciliary ganglion

Werirb1		retina
Hcfaa	heart	atrioventricular node
		cardiac myocytes
		heart
Hrgec	kidney	kidney
Hpf	lung	fetal lung
Nhbera		lung
Wi38		
Monocd14ro1746	monocytes	cd14+ monocytes
Hsmm	muscle	skeletal muscle
Hsmmt		smooth muscle
Lhcnm2		
Lncap	prostate	prostate
Prec		
Gm04503	skin	skin
Gm04504		
Nhdfad		
Nhek		
Rpmi7951		
Hasp	spine	dorsal root ganglion
		spine
		superior cervical ganglion
		trigeminal ganglion
Nt2d1	testis	testis
		testis germ cell
		testis intersitial
		testis leydig cell
		testis seminiferous tubule

Supplementary Table 4. Genes targeted by the enhancer cluster in Figure 6 (see legend in Figure 6 for more information).

- * transport
- * signal transduction
- * nucleocytoplasmic transport
- * embryo development
- * cell death
- * cell differentiation
- * cell signaling
- * anatomical structure formation involved In morphogenesis
- * cell proliferation
- * transmembrane support

Gene Symbol	Gene Description	GO Slim Terms									
STK38L	protein_kinase_activity		*								
SSPN	cell_junction										
DIP2B	transcription_factor_binding										
PPM1H	catalytic_activity										
KITLG	signal_transduction		*		*	*	*			*	

KCNA5	transmembrane_transport	*						*			*
PLEKHA5	phospholipid_binding										
WNK1	protein_kinase_activity	*	*				*				
ADAMTS20	proteolysis		*			*	*				
SRGAP1	signal_transduction		*				*		*		
CCDC91	protein_transport	*									
IFNG	cytokine-mediated_signaling_pathway	*	*	*		*	*	*	*	*	
BTBD11	DNA_binding										
TMTC2	endoplasmic_reticulum										
E2F7	regulation_of_transcription_DNA-dependent		*		*		*		*	*	
CDK17	protein_kinase_activity										
PPTC7	metal_ion_binding										
ETNK1	ATP_binding										
VEZT	cell_junction				*						
PRICKLE1	transcription_factor_binding	*	*	*	*		*		*		
CALCOCO1	signal_transduction		*								
LIMA1	cell_junction										
IFT81	cell_differentiation						*				
SYT1	cell_junction	*						*			
PTPRQ	receptor_activity				*		*		*		
CACNA1C	transmembrane_transport	*					*	*	*		
ERC1	Golgi_membrane	*	*								
KRR1	RNA_binding										
TMEM117	integral_to_membrane										
AEBP2	regulation_of_transcription_DNA-dependent										
DRAM1	apoptotic_process					*					
NUDT4	intracellular_signal_transduction	*	*	*							
EPS8	signal_transduction		*							*	
IFLTD1	cell_proliferation									*	
ANO6	ion_transport	*									*
DDX47	ATP_binding					*					
SLC6A15	transmembrane_transport	*									*
HPD	Golgi_membrane										
PTHLH	Golgi_apparatus		*				*	*	*	*	
IGF1	signal_transduction	*	*	*		*	*		*	*	
STAB2	receptor_activity	*							*		
EEA1	membrane_fraction	*						*			
C1R	proteolysis										
TMEM119	integral_to_membrane										
TSPAN11	membrane										
PPFIA2	receptor_activity										
NCOR2	negative_regulation_of_transcription_		*								
ATP2B1	transmembrane_transport	*									*
MLXIP	regulation_of_transcription_DNA-dependent	*		*							
GLIPR1L2	integral_to_membrane										
EPYC	extracellular_region										
PPP1R12A	signal_transducer_activity	*	*	*							
AMIGO2	cell_adhesion					*					
FAR2	endoplasmic_reticulum_membrane										
BICD1	transport	*	*								
NUAK1	protein_kinase_activity									*	
SLC38A2	transmembrane_transport	*						*			*
CRADD	signal_transduction		*			*					
EP400	nucleotide_binding										
DYRK2	protein_kinase_activity	*	*	*		*					
DCN	extracellular_space										
ZNF664	regulation_of_transcription_DNA-dependent										
SLC41A2	transmembrane_transport	*									*
HMGA2	negative_regulation_of_transcription_	*			*	*	*	*	*	*	
PDE3A	signal_transduction		*			*	*				

CHST11	transferase_activity		*		*	*	*		*	*	
PLEKHG6	phospholipid_binding		*								
TMTC3	integral_to_membrane										
ANO4	ion_transport										
NAV3	ATP_binding										
SLC38A4	transmembrane_transport	*									*
ANKS1B	cell_junction										
C12orf70	integral_to_membrane										
PLCZ1	intracellular_signal_transduction	*	*								
HCAR1	G-protein_coupled_receptor_activity										
CKAP4	perinuclear_region_of_cytoplasm										
USP15	proteolysis		*								
ITPR2	transmembrane_transport	*	*					*			
TBX3	negative_regulation_of_transcription_	*			*	*	*	*	*	*	
PTPRR	receptor_activity				*		*				
WNT5B	receptor_binding		*		*		*		*		
TSPAN8	signal_transducer_activity		*								
ST8SIA1	Golgi_membrane									*	
RASSF9	signal_transduction	*	*								
TSFM	intracellular										
TEAD4	regulation_of_transcription_from_RNA_pol_II_promoter		*		*		*		*		
TMEM132B	integral_to_membrane										
PHLDA1	regulation_of_transcription_from_RNA_polII_promoter					*					

Supplemental Notes and Results

1. Incorporating TF gene expression in assessing motif co-occurrence among correlated enhancer pairs.

In result section 5, we sought evidence of co-regulation by comparing the frequency of co-occurrence for each motif in correlated enhancer pairs to its expected frequency. To make these tests more conservative, instances of co-occurrence in a pair were only counted when there was at least one tissue in which both pair members were active and the cognate TF expressed. Motifs were not considered for which binding TF information was not available or that bind to TFs coded for by two or more genes. Approximately one-half (509) of the 981 motifs qualified. TFs were considered expressed in a given tissue if the normalized tag count density exceeded 0, where 0 was chosen due to the lack of any discontinuity in the distribution of tag count densities. (Based on this criterion, on average < 30% of TFs are expressed in each tissue). Under these conditions, there were a total of 67 motifs that co-occurred significantly more often than expected (FDR 5%, based on p-values from Fisher Exact Test) and present in at least 20 pairs, compared to zero motifs that occurred more often than expected in uncorrelated pairs. 20 of the 52 motifs previously found to co-occur out of 981 motifs were among the set of 67, in spite of the reduced test set of motifs. When thresholds of expression higher than 0 were used similar, if fewer, sets of significant motifs resulted (while still no motifs in random pairs

significantly co-occurred).

2. Correlated enhancer clusters share common regulatory motifs

We extended the pair-wise motif co-occurrence analyses to clusters of correlated enhancers. Disjoint clusters with at least 10 enhancers were greedily identified such that mean MI for all pairs within the cluster was at least 0.2 (other thresholds do not change the conclusion). Each TRANSFAC motif was assessed for enrichment in each cluster relative to other clusters based on a Fisher Exact Test, and significance was corrected for multiple testing. At a FDR threshold of 5%, for the 415 clusters, there were 44 instances of cluster-specific enrichments. In contrast, for a background set of 415 clusters using randomly chosen enhancers (mean pairwise *I* within a cluster << 0.1) sampled to match total motif occupancy, mean GC content, and the cluster size of the foreground, there were only 2 instances of cluster-specific enrichment.

3. Correlated enhancer pairs are potentially co-regulated

Co-regulated enhancers tend to share common motifs {Berman, 2004 #7}. To investigate whether the enhancer pairs with correlated activity are potentially co-regulated, next we tested whether correlated enhancers share significantly greater numbers of motifs than expected. We quantified motif overlap between the two enhancers using Jaccard index, defined as the ratio of the sizes of the intersection and the union of the two motifs sets. Separately for each distance-bin we compared Jaccard index values for the highly correlated enhancer pairs with those for pairs in the background using a Wilcoxon rank-sum test. The foreground and the background enhancer pairs were selected as for Result section 5 above. We found that in every distance bin the foreground pairs have a significantly greater fraction of shared motifs, with p-values ranging from 1.6e-04 to 6.1e-33 (Table 3a). The result remains highly significant when we repeated the analysis at the level of motif clusters instead of individual motifs (see M&M). As expected, the difference between foreground and background is amplified when only 52 significantly co-occurring motifs (section 5) were used to calculate Jaccard index (Table 3b). These results suggest that enhancer pairs highly correlated in their chromatin state share multiple motifs and are likely to be co-regulated.

4. Presence of shared motifs is predictive of enhancer DHS correlation

We assessed, using machine learning, whether the presence of common motifs can predict correlated activity of a pair of enhancers. For each enhancer pair we assigned one attribute per motif. The value of

the attribute was set to 1 if both enhancers had a motif instance and 0 otherwise. We then trained and tested a support vector machine (SVM) to discriminate between the foreground (FDR 0.01% was used for computational tractability) and the background enhancer pairs, using 10-fold cross validation. When using all 981 motifs as attributes, the SVM achieved an overall average classification accuracy of 73%. Importantly, there was very little reduction in performance (70%) when the model used only the 52 significantly co-occurring motifs (section 5). However, when we used 52 random motifs, the SVM accuracy was reduced to 55%, not much greater than random expectation of 50%. This result suggests that shared occurrence of a specific set of motifs is predictive of correlated enhancer activity.

5. Interactions between enhancer motifs and chromatin modification enzymes

To further probe the potential involvement of chromatin modification enzymes (CME) in regulating correlated enhancer activities, we assessed CMEs for their preferential interactions with the 52 motifs (Table 1) that significantly co-occur in correlated enhancers. The 52 motifs mapped to 146 unique proteins using TRANSFAC and ENSEMBL databases, while the remaining motifs mapped to 2227 proteins. There are more proteins than motifs due to ambiguous mapping of motifs to isoforms. A list of 828 CMEs was extracted from ENSEMBL database (version 67) based on GO term 'chromatin modification'. Protein-protein interactions were obtained from STRING database using the 'experimental' track. We assessed each of the 828 CMEs for preferential interaction with 146 TFs corresponding to significant motifs relative to the other 2227 TFs, using a Fisher Exact test, followed by multiple testing correction. At FDR = 5% we detected 28 CMEs to preferentially interact with significant TFs (Table 2). In contrast, there was no CME that preferentially interacted with non-significant TF. This result is especially interesting given that overall, the 146 significant TFs do not interact with CMEs any more than the other 2227 TFs.

6. Targets of correlated enhancer clusters have correlated expression and shared function

Next we extended our analyses in section 9 to clusters of correlated enhancers. We identified clusters of five or more enhancers that were mutually correlated (various thresholds from 0.2 to 0.5 were used), while enriched for at least one of the previously identified significantly enriched motif cluster. For each enhancer cluster a control cluster was created from non-correlated enhancers that mirrored the former's size and genomic footprint (i.e. intra-cluster genomic distances). As was true for correlated enhancer pairs, putative targets of correlated clusters (*i.e.*, the set of genes nearest to each enhancer), were more highly correlated in their normalized RNA-seq transcript counts than were background clusters. For each triplet of thresholds for (i) minimum cluster size (5-20), (ii) minimum pairwise I (0.2-0.5) within a cluster, and (iii) minimum fraction of cluster members (0.7-0.8) harboring the most

enriched meta-motif, the genes targeted by enhancers in clusters had higher Spearman correlation of transcription levels than the matching set of background enhancer clusters. For each parameter triplet, we compared the foreground and background for mean pair-wise correlation of expression within clusters. For the entire range of parameters, mean expression correlation within foreground clusters was consistently greater that for corresponding expression correlations within background clusters. Due to the variability in cluster counts for different parameters, p-values ranged from 0.02 to 4.1e-15 (Wilcoxon rank-sum test). These results suggest that gene targets of correlated enhancer clusters with shared motifs are co-expressed and presumably co-regulated.

Next we assessed enrichment of GO biological processes amongst the targets of an enhancer cluster using R's GOstats package. Enhancer clusters also revealed consistently greater GO functional enrichment than the background clusters. Across 10 parameter settings, the ratio of enriched GO terms (at FDR 0.01) per foreground cluster to enriched GO terms per background cluster ranges from 1.3-fold to 4.8-fold. On average, there is almost 3-fold higher GO term enrichment in the foreground (19.1 terms per cluster). When the FDR threshold is set to ~0 (i.e., $p < 1e-8$), there is 5-fold higher enrichment, on average, in the foreground (7.5 terms per cluster). As an example, for the parameter setting with the greatest fold enrichment of GO terms, the enriched terms are shown, separated by cluster, in Supplementary Table 3. These terms are consistently revealed across all parameters settings. Together, the GO enrichment and gene expression results illustrate that co-expression of genes with shared function is coordinately regulated across tissues by enhancers that share motifs and are epigenetically correlated across the same tissues.
